# Health Status in Informal Urban Settlements of Barishal, Bangladesh: Social Determinants, Environmental Risks, and Treatment‐Seeking Behavior as Structural Drivers

**DOI:** 10.1002/hsr2.73157

**Published:** 2026-09-01

**Authors:** Md. Ohidur Zaman, Farjana Afroz, Nahida Sultana, Dipti Ranjan Sahu

**Affiliations:** ^1^ University of Barishal Barishal Bangladesh; ^2^ University of Lucknow Lucknow Uttar Pradesh India

**Keywords:** health status, informal settlements, social determinants of health, treatment‐seeking behavior, urban health inequalities

## Abstract

**Background:**

Rapid urbanization in low‐ and middle‐income countries has intensified health inequalities within informal settlements, yet empirical analyses often isolate behavioral factors from structural determinants. In Bangladesh, unplanned urban growth has produced vulnerable settlements where social inequalities intersect with infrastructural deficits, particularly in secondary coastal cities such as Barishal, a climate‐sensitive region characterized by migration, poverty, and weak municipal capacity.

**Aim:**

This study examines the socio‐demographic and socioeconomic patterning of self‐reported health status among households residing in informal settlements in Barishal, Bangladesh, and assesses whether treatment‐seeking behavior independently influences health outcomes or whether structural determinants play a more significant role.

**Methods:**

A cross‐sectional quantitative research design was employed with 151 households selected through systematic random sampling from the Palashpur informal settlement under the Barishal City Corporation. Data were collected through face‐to‐face structured interviews using a pre‐tested questionnaire. Health status was measured using the Veterans RAND 12‐item composite index. Drawing on the Social Determinants of Health framework, we analyzed the effects of age, gender, education, income, household size, and treatment‐seeking behavior using correlation and multiple linear regression models.

**Results:**

The regression model explains 46.3% of the variance in health status (adjusted *R* ^2^ = 0.444, *p* < 0.001). Age emerges as the strongest predictor (*β* = −0.567, *p* < 0.001), followed by years of schooling (*β* = 0.213, *p* = 0.002) and household income (*β* = 0.159, *p* = 0.015). Gender becomes significant after controlling for structural variables (*β* = −0.197, *p* = 0.005), while treatment‐seeking behavior shows no independent association with health status. Environmental assessment revealed severe infrastructural deprivation: 62.3% share water sources with over 20 households, 88.7% share latrines with 10 or more households, 76.2% report poor or no drainage, and 90.1% receive no municipal waste collection.

**Conclusion:**

Findings indicate that health disparities in informal settlements are primarily structured by demographic positioning and socioeconomic resources rather than individual treatment choices. The study contributes localized evidence from a secondary coastal city and underscores the centrality of structural determinants in shaping urban health inequalities.

## 1 Introduction

Rapid urbanization in low‐ and middle‐income countries (LMICs) has generated extensive informal settlements characterized by overcrowding, environmental degradation, and fragile access to health services [1]. In Bangladesh, urban growth has been particularly unplanned, producing structurally vulnerable settlements where social inequalities intersect with infrastructural deficits [2, 3]. Barishal, a climate‐sensitive coastal city, exemplifies this pattern, where migration, poverty, and weak municipal capacity converge [4]. Within such settings, health status cannot be reduced to biomedical conditions; instead, it emerges from the interaction of socio‐demographic inequalities, environmental risks, and healthcare access constraints embedded in broader structural contexts. Despite significant improvements in national health indicators, intra‐urban disparities remain stark in Bangladesh [5]. Residents of informal settlements experience disproportionate exposure to contaminated water, inadequate sanitation, poor drainage, and limited waste management systems—conditions closely linked to communicable and chronic diseases [6]. However, policy responses often frame health vulnerability as an individual or behavioral issue, overlooking structural determinants. Furthermore, treatment‐seeking behavior in slum communities is shaped by economic precarity, spatial marginalization, and institutional mistrust [7]. There remains insufficient localized evidence from secondary coastal cities, such as Barishal, that integrates socio‐demographic, environmental, and healthcare access dimensions into a unified structural analysis.

Urban health scholarship increasingly emphasizes the structural production of health inequalities. The WHO Commission on Social Determinants of Health argued that health inequities arise from “the conditions in which people are born, grow, live, work and age” [8]. Empirical research across LMICs confirms that education, income, and gender significantly shape self‐reported health and morbidity outcomes [9, 10]. Urban slums are marked by entrenched socio‐demographic inequalities that shape health risks, access to resources, and overall well‐being. These settlements are characterized by deficits in education, precarious employment, income instability, and limited legal recognition, which collectively structure patterns of vulnerability [11, 12]. Empirical evidence from South Asia and sub‐Saharan Africa indicates that educational attainment remains substantially lower among slum populations than among formal urban residents, with pronounced gender disparities disadvantaging women and girls [13, 14]. Limited schooling constrains access to formal employment and income‐generating opportunities, reinforcing intergenerational cycles of poverty and poor health [15]. A study of 482 slum dwellers in Dhaka revealed critically low HIV transmission knowledge, with an average correctness of only 18.5%. Knowledge was positively associated with being male, higher education, higher income, and social media use, while smoking negatively affected knowledge [16]. Gender inequalities further mediate access to healthcare and health‐related decision‐making. In many slum contexts, men retain control over household finances and treatment expenditures, often marginalizing women's health needs or delaying care‐seeking [14, 17]. Women frequently navigate restricted mobility, social stigma, and financial dependence, which together undermine timely access to formal health services [9, 18]. Age compounds these disparities. Older adults in slum settings face disproportionately high levels of multimorbidity, with advancing age associated with increased risk of chronic conditions that erode functional capacity and quality of life [13, 19].

Migration status is another defining feature of slum demographics. Many settlements originate from rural‐to‐urban migration or environmental displacement triggered by disasters and climate‐related shocks, resulting in communities characterized by insecure tenure and “permanent temporariness” [20, 21]. Legal exclusion produces differentiated access to municipal services, as non‐notified settlements often lack formal recognition and are systematically excluded from infrastructure provision [22]. Socioeconomic gradients persist even within slums; households with stable or formal employment exhibit relatively better health outcomes than those reliant on irregular, informal labor [15, 23]. Multidimensional vulnerability assessments demonstrate significant intra‐urban variation, underscoring that slum populations are heterogeneous rather than uniformly deprived [11, 24].

Environmental deprivation is a defining characteristic of slum settlements, where inadequate housing, poor sanitation, limited water supply, and hazardous geographic locations converge to produce cumulative health risks [2, 21, 25]. In many African and Asian cities, waste disposal systems are either absent or severely underdeveloped, leading to widespread dumping of refuse in open spaces and water bodies. Such practices foster vector‐borne diseases and environmental contamination that extend beyond slum boundaries [25, 26]. Access to water and sanitation remains a critical determinant. Research in *Social Science & Medicine* and *The Lancet Global Health* indicates that unimproved sanitation and poor drainage are strongly associated with diarrheal disease, vector‐borne infections, and child mortality [27]. Recent evidence from Indian slum settlements shows that positive WaSH practices improve well‐being, while water insecurity harms it. Notably, having a toilet in slum households is linked to significantly better well‐being and sanitation‐related quality of life [28]. Also, research in Mumbai shows that households in non‐notified settlements consume substantially less water per capita than households in legally recognized neighborhoods, despite their geographical proximity [22]. Denial of formal water connections compels reliance on informal vendors, increasing costs and imposing disproportionate financial burdens on already marginalized households [22, 29].

Urban scaling analyses reveal that while slums in larger metropolitan areas may benefit from relatively better service provision than those in smaller cities, disparities between slum and non‐slum neighborhoods remain substantial [24]. The spatial marginalization of slums—often located on riverbanks, railway margins, steep slopes, or landfill sites—heightens exposure to flooding, landslides, and other environmental hazards [12, 20]. Recurrent flooding in low‐lying settlements contributes to the spread of waterborne diseases and repeated displacement [20, 30]. Systematic reviews confirm that these disparities are widespread across low‐ and middle‐income countries, highlighting the structural roots of health inequities in marginalized urban environments [31, 32]. Similarly, Lilford et al. [6] argued that slum health disadvantages stem not merely from poverty but from systemic failures in urban governance and planning. These deficits create “structural vulnerability,” wherein health risks are embedded in spatially segregated and politically neglected environments.

Economic precarity, gender norms, and fragmented service delivery systems shape health‐seeking behavior in slums. Health is frequently conceptualized as a means of sustaining work capacity rather than as an intrinsic right, leading residents to prioritize rapid symptom relief over comprehensive care (Patra and Bandyopadhya [23]). Treatment‐seeking behavior literature demonstrates that healthcare utilization in slum communities is rarely linear or timely [33]. Instead, individuals navigate pluralistic health systems involving informal providers, pharmacies, traditional healers, and public clinics [7]. In particular, pharmacies commonly serve as the first point of contact due to their proximity, affordability, and minimal waiting times—factors critical for daily wage earners [34, 35]. This reliance reflects structural necessity rather than preference [14, 36]. Recent studies highlight poor healthcare access among marginalized groups: Only 38.3% of slum‐dwellers in Iran with Type 2 diabetes utilized outpatient services [37], while in Northern Ghana, 44.1% of households used antibiotics without prescriptions, often obtained from informal sources [38]. Similarly, Kruk et al. [39] emphasized that poor‐quality healthcare contributes significantly to excess mortality in LMICs, highlighting institutional dimensions beyond simple availability. Paradoxically, even where service availability is relatively higher than in rural areas, outcomes remain poorer due to inequities in affordability, quality, and continuity of care [26, 30].

Healthcare fragmentation compels residents to navigate multiple providers, often leading them to engage in trial‐and‐error treatment sequences [35, 40]. Insurance coverage significantly increases the likelihood of timely care‐seeking, while perceived quality, waiting times, and provider attitudes influence service utilization [17, 34]. Despite geographic proximity to facilities, hidden costs and mistrust frequently deter engagement [35]. Spatial marginalization further complicates access. Informal settlements are often excluded from formal infrastructure planning, resulting in longer travel distances to public facilities and reduced emergency response times [41].

Gendered pathways to care are evident. Women often seek informal providers due to cultural familiarity, confidentiality, and reduced stigma, particularly in matters of reproductive health [14, 42, 43]. Men, conversely, may prefer formal facilities to ensure swift recovery and minimize income loss [14, 15]. These patterns mirror broader gendered divisions of labor and responsibility within households [17]. Social networks strongly influence treatment decisions. Word‐of‐mouth referrals and communal deliberation shape provider choice, sometimes perpetuating misinformation and delaying effective care [14, 35]. For instance, in urban Bangladesh, treatment delays may reflect strategic household calculations balancing cost, severity, and trust in providers [44]. Repeated unsuccessful treatment attempts deplete household resources and exacerbate vulnerability, reinforcing cycles of ill‐health and poverty [20, 34].

This study is theoretically grounded in the Social Determinants of Health [8] framework, which interprets health inequalities as results of disparities in access to material, educational, and economic resources. It emphasizes that demographic and socioeconomic factors shape risk exposure, resilience, and healthcare access. In informal urban settlements, factors such as educational attainment, income, household composition, and gender roles significantly affect health [2, 6]. Education enhances health literacy and navigational skills, while income improves nutrition, housing quality, and medical care. Age reflects biological vulnerability and cumulative disadvantage, and gender affects resource access and healthcare decision‐making. In addition, this study examines treatment‐seeking behavior through Kleinman's [45] framework to understand social decision‐making processes. This study assesses whether treatment choices directly influence health status or if socioeconomic positioning plays a more significant role, ultimately framing health status as a socially produced outcome shaped by demographic and socioeconomic factors rather than individual behaviors.

Existing studies on slum health frequently emphasize healthcare utilization and behavioral adaptation without adequately examining whether these behaviors independently shape health outcomes, even after structural inequalities are taken into account. Moreover, empirical evidence from secondary climate‐sensitive cities remains limited. Climate‐sensitive regions like Barishal experience seasonal flooding and drainage congestion, compounding sanitation risks [46, 47]. The intersection of climate exposure, infrastructural deficits, and healthcare access constraints remains underexplored within integrated structural frameworks. This study argues that health disparities are primarily driven by socio‐demographic positioning rather than treatment‐seeking behavior and focus on three following objectives: (1) to examine the relationship between socio‐demographic characteristics (age, education, income, gender) and self‐reported health status in informal settlements; (2) to assess environmental and infrastructural deficits (e.g., water access, waste management) impacting health outcomes; and (3) to investigate treatment‐seeking patterns and perceived barriers to modern healthcare. By integrating these factors, the study contributes to urban health sociology and public health policy, providing evidence for municipal planning and equity‐focused interventions aligned with the sustainable development goals.

## 2 Methodology

### 2.1 Study Design and Setting

This study employed a cross‐sectional quantitative research design to examine the socio‐demographic and socioeconomic determinants of health status in informal urban settlements. The research was conducted in the Palashpur slums under the Barishal City Corporation, Bangladesh. Palashpur is a densely populated informal settlement characterized by infrastructure limitations, insecure housing conditions, and limited access to formal healthcare [46]. According to local administrative estimates, the settlement comprises approximately 6,149 residents across 1819 households. The site was selected for its high population density, socioeconomic vulnerability, and relevance to urban health inequalities in a secondary coastal city context.

### 2.2 Sampling and Sample Size

The unit of analysis for this study was the household. According to the pilot survey, the Palashpur informal settlement comprises approximately 1819 households, with a total population of 6149. The required sample size was calculated using the Cochran formula for finite populations. Given logistical and field access constraints, the study adopted a 7.5% margin of error at a 95% confidence level. Assuming maximum variability (*p* = 0.5) to ensure a conservative estimate, the initial sample size (*n*_0_) was calculated using Cochran's formula, where *Z* = 1.96 (95% confidence), *p* = 0.5, and *e* = 0.075. After adjusting for the finite population (*N* = 1 819 households), the corrected sample size was 157 households.

Field data collection resulted in 157 completed interviews; however, six questionnaires were excluded due to substantial missing or incomplete responses, yielding a final analytical sample of 151 households. This final sample remains statistically adequate for multivariate regression analysis, satisfying the recommended minimum observation‐to‐predictor ratio and providing sufficient statistical power to detect medium effect sizes.

Households were selected using systematic random sampling. A sampling interval was determined by dividing the total number of households (1819) by the required sample size (157), producing an interval of approximately 12. A random starting point was selected, and every 12th household was approached for participation.

### 2.3 Data Collection Procedures

Data were collected through face‐to‐face structured interviews using a pre‐tested questionnaire. The survey instrument was administered to the household head or the second‐most‐influential household member after the head. Data collection took place over 4 weeks, from September 10 to October 10, 2025, which is typically the end of the wet season. Enumerators (post‐graduate students from the Department of Sociology) were trained to ensure consistent delivery of questions and minimize interviewer bias. Participation was voluntary, and informed consent was obtained prior to data collection. The approximate average duration of each interview was around 35 min.

### 2.4 Measures

#### 2.4.1 Dependent Variable: Health Status

Health status was measured using the Veterans RAND 12‐item composite index [48, 49, 50], capturing self‐reported physical functioning, daily activity limitations, emotional well‐being, social functioning, and general health perception. Items included mobility limitations (e.g., walking 2 km, climbing stairs), bodily pain, emotional states (e.g., feeling calm, happiness, feeling downhearted), and overall health rating. Responses were coded and summed to generate a continuous health status score, with higher scores indicating better health. This scale was developed in 1997 by Dr. Lewis E. Kazis and his research team [51] at Boston University School of Public Health.

#### 2.4.2 Independent Variables

The key independent variables included:

- Age (continuous, in years)
- Sex (dummy coded: 0 = male, 1 = female)
- Years of schooling (continuous)
- Monthly household income (continuous, in Bangladeshi Taka)
- Household size (number of family members)
- Treatment‐seeking behavior (categorical variable capturing the first treatment source during illness).

These variables were selected based on the Social Determinants of Health framework and prior literature on urban health inequalities.

### 2.5 Statistical Analysis

Data were analyzed in several stages using SPSS. First, descriptive statistics were calculated to summarize demographic characteristics and health indicators. Second, bivariate associations were examined using Pearson correlation for continuous variables and *χ* ^2^ tests for categorical variables. Third, multiple linear regression analysis was conducted to estimate the independent effects of socio‐demographic and socioeconomic predictors on health status. The model included age, sex, education, income, and household size as predictors. Treatment‐seeking behavior was examined separately through cross‐tabulation and *χ* ^2^ analysis. Model diagnostics included assessments of multicollinearity using variance inflation factors (VIFs), normality of residuals, and homoscedasticity. Statistical significance was set at *p* < 0.05.

### 2.6 Ethical Considerations

The study was conducted in accordance with the ethical principles outlined in the Declaration of Helsinki for research involving human participants. Participation was voluntary, and informed consent was obtained from all participants. They were made aware of their rights and assured that their responses would remain anonymous. Data were securely stored and used solely for research purposes, in compliance with confidentiality guidelines. The authors affirm that all research procedures complied with institutional ethical standards and relevant national guidelines for social science research involving human participants. While generative AI tools and editing software were utilized to improve clarity and grammar, all research decisions and interpretations were made by the author, who is committed to maintaining the manuscript's integrity and originality.

### 2.7 Data Analysis and Presentation

The socio‐economic characteristics of urban slum dwellers reveal a structurally vulnerable population characterized by gender imbalance, low levels of education, and constrained income (Table 1). Two‐thirds of respondents were female (66.2%), indicating that women disproportionately participate in day‐to‐day caregiving and health management activities within slum households. The age distribution clustered mainly between 25 and 45 years, representing the economically active population, yet this demographic advantage is offset by low human capital. A striking 58.3% of respondents had no formal schooling, while only 9.2% completed more than 6 years of education. Such educational deprivation directly limits health literacy, negotiation capacity with providers, and understanding of preventive care. Income distribution further reflects precarity: 43.7% of households survive on ≤ 5000 BDT per month, and only 19.2% report earnings above 9000 BDT. Considering average urban living costs, the majority are below subsistence levels. Family structure intensifies economic strain—63.6% live in households with 5–8 members, and 45% have three or more children. High dependency ratios combined with irregular income reinforce chronic vulnerability. Overall, the socio‐economic profile demonstrates intersecting disadvantages—gendered caregiving burdens, limited schooling, overcrowded households, and poverty—which collectively shape health‐seeking decisions and overall health outcomes among slum residents.

**Table 1:** Socio‐economic profile of respondents (*N* = 151).

Variable	Category	*n*	%
Sex	Male	51	33.8
	Female	100	66.2
Religion	Muslim	119	78.8
	Hindu	32	21.2
Education (years)	No schooling	88	58.3
	1–5 years	49	32.5
	6–9 years	14	9.2
Monthly family income (BDT)	≤ 5000	66	43.7
	6000–8000	56	37.1
	≥ 9000	29	19.2
Number of children	0–2	83	55.0
	3–6	68	45.0
Family size	2–4 members	52	34.4
	5–8 members	96	63.6
	9+ members	3	2.0

### 2.8 Environmental and Infrastructural Features

Environmental conditions reflect severe infrastructural deprivation (Table 2). Although 100% of households rely on tube wells for drinking water, access is intensely shared: 62.3% report that more than 20 households use the same water source, raising contamination risks. Sanitation is equally strained; only 27.8% use well‐built latrines, while 21.2% practice open defecation. Furthermore, 88.7% of latrines are shared by 10 or more households, indicating extreme overcrowding and compromised hygiene. Drainage systems are critically inadequate—76.2% report either poor or no drainage, contributing to stagnant water and increased risk of vector‐borne disease. Waste management is nearly absent; 70.2% dispose of garbage in non‐designated places, and 90.1% report that municipal collection services never visit their area. These infrastructural deficits create a structural disease ecology characterized by environmental exposure, recurrent infections, and chronic stress. The spatial marginalization of slum settlements—marked by overcrowding, inadequate sanitation, poor drainage, and the absence of municipal services—demonstrates that poor health outcomes are not merely individual issues but are deeply embedded in urban structural inequalities.

**Table 2:** Environmental and infrastructure conditions (*N* = 151).

Indicator	Category	*n*	%
Type of latrine	Well‐built	42	27.8
	Half‐built	77	51.0
	Open	32	21.2
Latrine sharing	≥ 10 households	134	88.7
Water source	Tube well	151	100
Water sharing	≥ 20 households	94	62.3
Drainage	Poor/None	115	76.2
Garbage disposal	No fixed place	106	70.2
City corporation collection	None	136	90.1

### 2.9 Health‐Seeking Behavior of Poor People (Last 3 Months)

Health‐seeking behavior demonstrates a pattern of informalization and economic constraint (Table 3). Over half of respondents (53.0%) practice self‐medication at least occasionally, and 57.0% take medicines based on advice from family or neighbors. Although 45.7% report choosing modern medicine, a slightly higher proportion (54.3%) relies on traditional medicine either exclusively or sequentially. Critically, 57.6% first seek treatment from unqualified providers, while only 20.5% initially consult professional doctors. This indicates that informal healthcare markets dominate primary access in slum contexts. Financial hardship is the principal driver of delay—70.9% cite lack of money as the main reason for postponing treatment. Spousal decision‐making power is mainly spousal (53.0%), reflecting intra‐household gendered authority structures. Preventive health behavior presents mixed patterns: While child vaccination coverage is high (83.4%), contraceptive use remains below half (47.7%). Cultural beliefs also intersect with biomedical practice: 33.8% believe illness is caused by the evil eye, and 17.2% attribute illness to spiritual causes. Together, these findings reveal pluralistic health behavior shaped by poverty, social networks, gender dynamics, and cultural belief systems. The dominance of informal providers and the delay in care suggest both economic exclusion and limited trust in formal health systems.

**Table 3:** Treatment‐seeking practices (*N* = 151).

Indicator	Category	*n*	%
Self‐medication	Yes/Sometimes	80	53.0
Medicine by others' suggestion	Yes/Sometimes	86	57.0
Type of medicine chosen	Modern only	69	45.7
	Traditional/Both/Sequential	82	54.3
First treatment source	Unqualified providers	87	57.6
	Professional providers	31	20.5
Decision maker	Spouse	80	53.0
Delay reason	Lack of money	107	70.9
Vaccination (children)	Yes	126	83.4
Contraceptive use	Yes	72	47.7

### 2.10 Barriers to Taking Modern Treatment

Economic cost is the most dominant barrier to modern healthcare utilization. Table 4 summarizes the barriers to seeking modern treatment. Nearly all respondents (98.0%) perceive modern medicine as high or very high in cost, and 94.0% explicitly identify cost as a barrier. Geographical constraints further compound exclusion; 66.9% report distance as a barrier, and 57.0% cite transport difficulties. Over one‐third describe transport as uncomfortable or very uncomfortable, reflecting infrastructural and mobility deficits. Institutional trust is also fragile: 56.3% believe provider behavior constitutes a barrier, and 80.1% perceive unnecessary diagnostic tests as exploitative. Although 93.4% report visiting nearby medical centers at some point, physical proximity does not eliminate access barriers due to cost and quality concerns. The fact that 57.6% initially seek unqualified providers illustrates adaptive behavior within constrained environments—slum dwellers prioritize affordability, immediacy, and familiarity over formal medical credentials. These barriers highlight a multidimensional exclusion framework: economic (cost), spatial (distance), institutional (provider behavior), and procedural (diagnostic expenses). Together, they create structural barriers that delay treatment and reinforce reliance on informal care markets.

**Table 4:** Perceived barriers to modern healthcare (*N* = 151).

Barrier	Category	*n*	%
Cost perception	High/Very high	148	98.0
Cost as barrier	Yes	142	94.0
Distance barrier	Yes	101	66.9
Transport barrier	Yes	86	57.0
Negative provider behavior barrier	Yes	85	56.3
Unnecessary tests barrier	Yes	121	80.1
First treatment from unqualified provider	Yes	87	57.6

### 2.11 Health Status

Table 5 presents the distribution of self‐reported physical and mental health indicators among respondents (*N* = 151). More than half of participants rated their general health as “fair” (54.3%), while nearly one‐quarter (24.5%) rated it as “poor,” suggesting substantial perceived health vulnerability within the sample. The self‐rated “fair” health category does not indicate the absence of health issues. Instead, due to their low‐income and marginalized circumstances, respondents often normalize chronic hardships, limited mobility, fatigue, or untreated conditions as part of their daily lives. As a result, they tend to rate their health as “fair” rather than “poor.” Physical functioning limitations were pronounced, particularly for walking 2 km, with 62.3% reporting being limited “a lot,” indicating restricted mobility. Over half (55.6%) experienced some degree of difficulty in daily activities due to physical health, and emotional limitations were similarly prevalent, with 72.2% reporting moderate to extreme difficulty. Social functioning appeared comparatively less restricted, although 26.5% experienced moderate to substantial limitations. Despite nearly half (49.0%) reporting no bodily pain, indicators of mental well‐being revealed notable psychosocial strain: 58.3% reported having little or no energy, 68.2% felt downhearted at least some of the time, and 27.8% experienced little or no happiness. Overall, the distribution suggests that while acute pain levels are relatively low, functional and emotional health burdens remain considerable, highlighting multidimensional health vulnerability that extends beyond purely physical symptoms and reflects broader psychosocial and structural health constraints.

**Table 5:** Distribution of self‐reported physical and mental health indicators (*N* = 151).

Domain	Category	*n*	%
General Health Status	Very good	1	0.7
	Good	31	20.5
	Fair	82	54.3
	Poor	37	24.5
Physical Functioning	Carrying 10 kg—Limited a lot	57	37.7
	Carrying 10 kg—Limited a little	94	62.3
	Climbing stairs—Limited a lot	44	29.1
	Climbing stairs—Limited a little	75	49.7
	Climbing stairs—Not limited	32	21.2
	Walking 2 km—Limited a lot	94	62.3
	Walking 2 km—Limited a little	50	33.1
	Walking 2 km—Not limited	7	4.6
Daily Activity Limitation (Physical)	None	26	17.2
	A little	41	27.2
	Some	84	55.6
Daily Activity Limitation (Emotional)	Not at all	10	6.6
	Slightly	32	21.2
	Moderately	45	29.8
	Quite a bit	39	25.8
	Extremely	25	16.6
Social Activity Limitation	Not at all	48	31.8
	Slightly	63	41.7
	Moderately	30	19.9
	Quite a bit	10	6.6
Bodily Pain	None	74	49.0
	Very mild	58	38.4
	Mild	15	9.9
	Moderate	4	2.6
Mental Well‐Being	Calm and peaceful—Little/None of time	65	43.0
	Energy—Little/None of time	88	58.3
	Downhearted—Some or more	103	68.2
	Happiness—Little/None of time	42	27.8

### 2.12 Treatment‐Seeking Behavior and Self‐Rated Health Status

The cross‐tabulation analysis examined the association between the type of first treatment sought during illness and respondents' self‐rated general health status (Table 6). Descriptively, the majority of respondents reported seeking treatment from unqualified providers (*n* = 87), followed by professional providers (*n* = 31). Among those reporting “poor” health, a notable proportion sought care from professional (*n* = 12) and traditional providers (*n* = 7). In contrast, respondents reporting “fair” health were more likely to rely on unqualified providers (*n* = 54). However, the Pearson *χ* ^2^ test indicated that the association between treatment‐seeking behavior and self‐rated health status was not statistically significant, *χ* ^2^ (12) = 17.47, *p* = 0.133. Thus, although variations in treatment patterns are descriptively observable, they do not demonstrate a statistically robust relationship with perceived health status. It is important to note that 60% of cells had expected counts below five, suggesting limited statistical power and caution in interpreting these categorical associations.

**Table 6:** Association between first treatment‐seeking behavior and self‐rated health status (*N* = 151).

**Treatment type**	**Very good**	**Good**	**Fair**	**Poor**	**Total**
Self	0	1	4	1	6
Unqualified provider	1	17	54	15	87
Para‐professional	0	3	9	2	14
Professional	0	9	10	12	31
Traditional	0	1	5	7	13
Total	1	31	82	37	151

*Note: χ* ^2^ (12) = 17.47, *p* = 0.133.

Further, Pearson correlation analysis revealed several statistically significant relationships among demographic, socioeconomic, and health‐related variables (Table 7). Health status was strongly and negatively correlated with age (*r* = −0.575, *p* < 0.001), indicating declining health with increasing age. In contrast, years of schooling showed a strong positive association with health status (*r* = 0.432, *p* < 0.001), reinforcing the educational gradient in health. Income demonstrated a modest but significant positive relationship with health status (*r* = 0.166, *p* = 0.042), while family size was negatively correlated (*r* = −0.211, *p* = 0.009), suggesting possible household resource constraints. The duration of treatment after illness was negatively associated with health status (*r* = −0.253, *p* = 0.002), suggesting that individuals with poorer health seek modern therapy later after they became ill. Additionally, age was positively correlated with family size (*r* = 0.439, *p* < 0.001) and negatively associated with years of schooling (*r* = −0.274, *p* < 0.001), highlighting structural demographic patterns within the sample. Overall, the correlation matrix supports a social determinants framework in which age, education, income, and household composition significantly shape health outcomes.

**Table 7:** Pearson correlation matrix of demographic, socioeconomic, and health variables (*N* = 151).

Variable	1	2	3	4	5	6
1. Age	1					
2. Schooling	−0.274***	1				
3. Income	0.035	0.135	1			
4. Family Size	0.439***	−0.074	0.299***	1		
5. Treatment Duration	0.214**	−0.107	−0.291***	0.159	1	
6. Health Status	−0.575***	0.432***	0.166*	−0.211**	−0.253**	1

* *p* < 0.05

** *p* < 0.01

*** *p* < 0.001 (two‐tailed).

Moreover, a multiple linear regression model was estimated to examine the independent effects of demographic and socioeconomic factors on household health status. The overall model was statistically significant, *F* (5, 145) = 24.97, *p* < 0.001, and explained 46.3% of the variance in health status (*R* ^2^ = 0.463; Adjusted *R* ^2^ = 0.444). This indicates a substantial explanatory power for the selected predictors (Table 8).

**Table 8:** Multiple linear regression analysis.

Predictor	*B*	SE	*β*	*t*	*p*
Sex	−3.103	1.091	−0.197	−2.844	0.005
Age	−0.365	0.048	−0.567	−7.589	<0.001
Years of schooling	0.602	0.191	0.213	3.145	0.002
Income	0.000	0.000	0.159	2.455	0.015
Family size	−0.221	0.307	−0.052	−0.718	0.474

*Note:* Model fit: *R* ^2^ = 0.463; adjusted *R* ^2^ = 0.444; *F* (5,145) = 24.97, *p* < 0.001

Age emerged as the strongest predictor of health status (*β* = −0.567, *p *< 0.001). Holding other variables constant, increasing age significantly reduced health scores, indicating age‐related vulnerability in health outcomes. Years of schooling were positively associated with health status (*β* = 0.213, *p* = 0.002), suggesting that education enhances health resilience and possibly access to health‐related knowledge and services. Monthly household income also showed a significant positive effect (*β* = 0.159, *p* = 0.015), supporting the social determinants of health framework, which posits that economic resources improve health outcomes. Interestingly, sex became statistically significant in the multivariate model (*β* = −0.197, *p* = 0.005), despite being non‐significant in the bivariate correlation. This suggests a suppression or interaction effect when controlling for age and socioeconomic factors, indicating gendered disparities in health once structural conditions are accounted for. However, Family size did not retain statistical significance in the multivariate model (*β* = −0.052, *p* = 0.474), suggesting that other demographic or socioeconomic factors may mediate its bivariate association with health status.

## 3 Result and Discussion

The findings provide strong empirical support for the social determinants of health perspective. Age represents biological and lifecycle vulnerability, while education and income function as structural resources that enhance health capacity. The gender effect in the adjusted model suggests that health inequalities may be embedded within broader socio‐demographic structures rather than operating independently. The relatively high adjusted explained variance (44.4%) indicates that demographic and socioeconomic characteristics substantially shape household health outcomes in the study context. While infrastructural indicators were not included in the multivariate regression model, the descriptive findings strongly suggest a hazardous living environment. This environment is characterized by overcrowded sanitation, inadequate drainage, and poor waste management. These conditions likely act as contextual factors that influence both exposure to disease and barriers to treatment.

Furthermore, this study aligns closely with the broader literature on the Social Determinants of Health while offering context‐specific insights from a secondary coastal city in Bangladesh. Consistent with the WHO Commission on Social Determinants of Health [8] and subsequent empirical studies [9, 10], age and socioeconomic positioning emerged as the most significant predictors of self‐reported health status. The strong negative association between age and health observed in this study supports life‐course vulnerability theories, which argue that cumulative exposure to structural disadvantage intensifies health decline over time. Similarly, the positive educational gradient identified here reinforces extensive evidence from LMIC contexts showing that education enhances health literacy, service navigation, and preventive behaviors. Research in Kampala's slums found that older age and lower education were linked to poorer self‐rated physical health, particularly among orphans, homeless youth, and those without basic utilities, who reported worse health outcomes [52]. Also, the positive association between household income and health status further corroborates findings from urban health research demonstrating that material resources mediate access to nutrition, sanitation, and quality healthcare [2, 6]. However, unlike some prior studies that emphasize environmental infrastructure as a dominant determinant of slum health, the present analysis highlights demographic and socioeconomic factors as the most robust predictors in the multivariate model. This suggests that while environmental deprivation remains critical, its effects may operate indirectly through socioeconomic positioning. In addition, a notable divergence from parts of the treatment‐seeking literature [7] is the absence of a statistically significant association between initial treatment choice and health status. The lack of a statistically significant link between treatment‐seeking behavior and self‐reported health status should not be seen as evidence that healthcare utilization is unimportant. Instead, this finding likely highlights the limited healthcare options available in informal settlements, where factors such as affordability, accessibility, and perceived provider quality shape treatment choices. In this context, variations in treatment pathways may have only a limited independent impact, as most residents face similar structural disadvantages. The dominance of unqualified providers, widespread treatment delays due to financial hardship, and perceived institutional barriers suggest that systemic limitations may, in turn, constrain healthcare effectiveness.

This study's contribution goes beyond simply reaffirming well‐established socioeconomic gradients in health. It demonstrates that, within a structurally deprived informal settlement within a secondary coastal city, demographic and socioeconomic factors have significantly more explanatory power than treatment‐seeking pathways alone. Instead of contradicting existing research, this finding refines it by highlighting the importance of structural explanations that focus on material deprivation and social positioning. Thus, the study strengthens structural interpretations of health inequality by empirically demonstrating that demographic and socioeconomic factors exert stronger explanatory power than healthcare pathway variation alone.

## 4 Limitations

This study has several limitations that should be considered when interpreting the findings. First, the research was conducted in a single informal settlement (Palashpur slum), which may limit the representativeness of the results for other urban slum contexts. Second, the cross‐sectional design captures associations at one point in time and therefore cannot establish causal relationships between the predictors and health outcomes. Third, health status was measured through self‐reported responses, which may be subject to recall bias and subjective perception bias. Fourth, although environmental and infrastructural conditions were descriptively documented, these variables were not incorporated into the multivariate regression model due to measurement and sample‐size constraints. Finally, the findings are most applicable to informal settlements with similar socioeconomic and environmental characteristics in coastal urban Bangladesh and should be generalized with caution to other settings.

## 5 Conclusion

This study examined the health status of slum dwellers in Palashpur, Barishal City, with particular attention to treatment‐seeking behavior and the socioeconomic, demographic, and environmental factors influencing health outcomes. The findings reveal that residents experience multiple forms of structural disadvantage, including poverty, low educational attainment, inadequate sanitation, poor drainage, overcrowded living conditions, and limited access to formal healthcare services, all of which contribute to health vulnerability. Also, this study found that treatment‐seeking behavior is strongly constrained by affordability, accessibility, and reliance on informal healthcare providers. While self‐medication, delayed treatment, and consultation with unqualified practitioners were common, treatment‐seeking pathways were not significantly associated with self‐reported health status, and in contrast, age, education, income, and gender emerged as significant predictors of health outcomes. These findings suggest that health inequalities within informal settlements are shaped more by structural socioeconomic conditions than by healthcare choices alone.

Guided by the Social Determinants of Health framework, this study contributes to urban health research by providing evidence from a secondary coastal city in Bangladesh, a context that remains underrepresented in the literature. The findings underscore the need for integrated policy interventions that address socioeconomic deprivation, environmental deficiencies, and barriers to quality healthcare. Improving sanitation, housing conditions, educational opportunities, and access to affordable healthcare services is essential for reducing health inequalities and promoting healthier and more resilient urban communities. Future research should adopt longitudinal designs to examine causal pathways and life‐course dynamics more robustly. Integrating environmental and infrastructural variables—such as sanitation access, housing quality, and climate exposure—into multivariate models would further clarify structural mechanisms. Additionally, mixed‐method approaches could explore how socioeconomic positioning interacts with cultural interpretations of illness and healthcare navigation. Expanding the analysis to comparative urban settings would also enhance generalizability and policy relevance.

## Author Contributions

**Md. Ohidur Zaman:** conceptualization, methodology, software, formal analysis, writing – original draft, data curation, visualization, resources, project administration. **Farjana Afroz:** investigation, conceptualization, data curation, writing – review and editing. **Nahida Sultana:** conceptualization, data curation, investigation, writing – review and editing. **Dipti Ranjan Sahu:** supervision, writing – review and editing, validation, methodology.

## Funding

The authors have nothing to report.

## Conflicts of Interest

The authors declare no conflicts of interest.

## Transparency Statement

The corresponding author, Dr. Md. Ohidur Zaman, confirms that this manuscript provides an honest, accurate, and transparent account of the reported study. All essential aspects of the research have been included, and any deviations from the original study plan (and, if applicable, its registration) have been fully addressed.

## Data Availability

The data that support the findings of this study are available from the corresponding author upon reasonable request.
